# Taravana syndrome and posterior reversible encephalopathy syndrome: a microbubble hypothesis for neurological accidents in breath-hold divers

**DOI:** 10.3389/fphys.2024.1478650

**Published:** 2024-09-24

**Authors:** Arnaud Druelle, Olivier Castagna, Romain Roffi, Pierre Louge, Anthony Faivre, Jean-Eric Blatteau

**Affiliations:** ^1^ Service de Médecine Hyperbare et d’Expertise Plongée (SMHEP), Hôpital d’Instruction des Armées Sainte-Anne, Toulon, France; ^2^ Equipe de Recherche Subaquatique et Hyperbare, Institut de Recherche Biomédicale des Armées, Toulon, France; ^3^ Unité Neurovasculaire, Hôpital d’Instruction des Armées Sainte-Anne, Toulon, France

**Keywords:** neurological symptoms, breath-hold diving, decompression sickness, bubble, MRI, Taravana, posterior reversible encephalopathy syndrome, underwater scooter

## Abstract

Breath-hold diving is a challenging activity that can lead to serious and dangerous complications, such as the “Taravana” syndrome. This syndrome is characterized by the onset of neurological symptoms after deep or repeated dives. The main clinical manifestations are cerebral, including stroke and cognitive impairment. The pathophysiology of Taravana syndrome is still widely debated, but the most accepted theory is that it is a specific form of decompression sickness. We have reviewed the main theories explaining the onset of Taravana syndrome and, through the description of a particularly illustrative case of a freediver using an underwater scooter, we have formulated a hypothesis according to which micro-bubbles formed directly in cerebral structures would be at the origin of this syndrome. MRI showed diffuse encephalopathy with vasogenic edema. Analysis of the radiological sequences did not suggest an ischemic or embolic mechanism. This finding is likely to be associated with the diagnosis of posterior reversible encephalopathy syndrome. The rapid ascent speeds associated with underwater scooter use could potentially result in the formation of nitrogen micro-bubbles in the capillaries of brain tissue. The emergence of scooters in freediving can be a hazard because of their ability to facilitate very rapid ascents. It is therefore essential to take preventive measures to ensure the safety of users of these devices.

## Introduction

Breath-hold (BH) diving, also known as freediving, is the sport of diving underwater without the use of a breathing apparatus. BH diving has a long history and cultural significance in many regions of the world, particularly in the Pacific Islands, where it is practised for subsistence, recreation and competition. BH diving poses significant challenges and hazards to the human body, particularly in terms of gas exchange, pressure regulation and hypoxia. A serious and dangerous complication of freediving is “Taravana” syndrome, which is considered to be a specific form of decompression sickness (DCS) (Bagnis, 1968; Cross, 1965; Paulev, 1965).

Taravana syndrome was first described in 1956 by Dr Truc, a French military doctor, who observed neurological disorders in mother-of-pearl fishermen in the Tuamotu Islands, a small archipelago in French Polynesia (Bagnis, 1968). He wrote an observational report, later published in 1968, in which he coined the term Taravana, which in the local dialect meant “to fall mad.” He attributed the symptoms to the formation of nitrogen bubbles in the brain, caused by repeated decompressions during ascent to the surface between breath-hold dives, constituting an authentic cerebral DCS (Bagnis, 1968).

The epidemiology of Taravana syndrome is largely unknown due to the difficulty of collecting and reporting meaningful measures. Most of the available data is based on anecdotal reports or small case series, which may not reflect the true incidence and prevalence of the condition. In addition, the diagnosis of Taravana syndrome is often difficult and subjective, and can vary depending on the criteria and methods used (Lemaître et al., 2009; Blogg et al., 2023).

According to the literature, Taravana syndrome is a rare condition, affecting only a small proportion of breath-hold divers performing certain dive profiles. The majority of Taravana cases are reported from tropical and subtropical regions, such as French Polynesia, Japan, Hawaii and the Mediterranean, where BH diving is more common and practised throughout the year. However, Taravana syndrome can occur in any geographical location and at any time of year, as long as the diving conditions and behaviour are conducive to the formation of bubbles. The most important factor in determining the risk of Taravana syndrome is the dive profile, which includes the number, depth, duration and frequency of dives, as well as surface intervals and ascent and decent rates. The more dives, the deeper, the longer and the faster the dives, and the shorter the surface intervals, the higher the risk of Taravana syndrome. These diving habits increase tissue nitrogen loading during descent and bottom time and decrease nitrogen unloading during ascent and surface time, leading to the formation of bubbles.

The attractiveness of extreme sports, of which freediving is now a part, and the ever-increasing depth limits reached, have led us to rethink the accidentology of freediving. Underwater scooters, also known as diver propulsion vehicles, are devices that allow divers to move faster and deeper in the water (Grandjean and Fanton, 1996). Underwater scooters are increasingly being used by breath-hold divers for a variety of purposes, including exploration, hunting and filming. However, underwater scooters also increase the risk of Taravana syndrome by allowing divers to make more frequent and longer dives, with shorter surface intervals and faster ascent and descent rates.

While the pathophysiology of Taravana syndrome is still widely debated, most authors consider that the onset of Taravana symptoms initially results from the presence of bubbles in the body, and more specifically in the brain caused by repeated decompressions during the ascent to the surface between breath-hold dives. DCS is usually considered to be specific to scuba diving, where the accumulation of nitrogen in the tissues depends on the time spent underwater and the depth reached. However, it has been shown that certain BH diving profiles can result in an inert gas load sufficient to promote DCS (Radermacher et al., 1992) and several studies have found the presence of circulating bubbles by Doppler in BH divers (Spencer and Okino, 1972; Lemaître et al., 2014; Cialoni et al., 2016; Barak et al., 2020).

However, the clinical presentation of Taravana syndrome is quite different from the clinical forms of DCS observed in diving (Bagnis, 1968). Taravana symptoms are predominantly cerebral, with pronounced symptoms of stroke, disproportionate agitation, and cognitive impairment of various and varying intensities that may go unnoticed and affect long-term neuropsychological health (Kohshi et al., 2014). On the other hand, neurological DCS in scuba diving mainly affects the spinal cord, with sensory or motor deficits in the limbs, without any cerebral symptoms (Blatteau et al., 2011).

There are currently two opposing views on the origin of the brain damage caused by bubbles in the Taravana. Some believed that the initial mechanism is the passage of air bubbles from the arterial side through a right-to-left shunt (Schipke and Tetzlaff, 2016), while others have proposed that bubble formation occurs directly in the brain in the form of autochthonous bubbles or cerebral arterial bubbles. The influence of other factors such as hypoxia, the immuno-inflammatory state, or apnea-related cardiopulmonary anatomical changes is also debated (Kohshi et al., 2021; Foster et al., 2016).

The aim of this article was to review and present the main theories underlying Taravana syndrome. From a very interesting case of Taravana, we wanted to highlight the magnetic resonance imaging (MRI) findings showing diffuse encephalopathy with vasogenic edema. These images clearly recalled the radiologic picture of posterior reversible encephalopathy syndrome (PRES). In our opinion, these data strongly suggested a mechanism of cerebral involvement by the *in situ* formation of autochthonous bubbles.

Furthermore, this case allowed us to illustrate the dangers of using an underwater scooter in freediving with a very high ascent speed, a factor that favoured the formation of bubbles, in line with the proposed pathophysiological hypothesis.

## Description of the case

A 45-year-old man presented to the Toulon Hyperbaric Centre with a loss of consciousness at the surface followed by a hypertonic seizure shortly after a deep breath hold dive. He had no medical history and was not taking any medication. He has been a regular deep freediver for several years. He recently acquired an underwater propulsion system that allowed him to descend to great depths with little effort, remain at the bottom and then ascend with minimal muscle effort to conserve oxygen and extend his freediving times.

**FIGURE 1:**
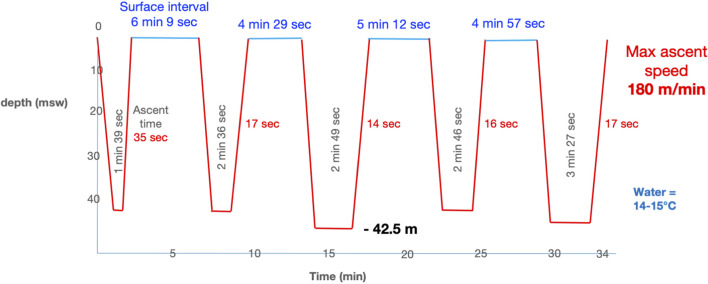
Freediving profile of the accident diver using an underwater scooter.

After warming up on the surface with a few freedives to a maximum depth of 10 m underwater, he made a series of 4 freedives to 41 msw, with surface intervals of 4 to 6 min and breath-hold times from 1 min and 39 s to 3 min and 27 s for the last (Figure 1). To descend and return to the surface, he used the underwater scooter.

On the surface, the breath-hold diver was greeted by a scuba diver who ensured the diver’s safety, particularly in the event of loss of consciousness. The team followed the safety protocol for the first 2 min on the surface. The diver then lost consciousness at the surface, with the snorkel in the mouth and no abnormal movements. The safety diver observed that the trismus made it difficult to remove the snorkel.

The diver was successfully rescued at 1:00 p.m. He was unconscious with generalised rigidity and trismus. He was evacuated by helicopter on high-flow oxygen therapy with a high-concentration mask. Upon arrival at the hyperbaric centre at 2:40 p.m., the casualty was confused (Glasgow score 13–14) with normal photopupillary reflexes. Higher functions were impaired with temporal disorientation, impaired immediate memory and difficulty in answering semi-complex questions. The general clinical examination was unremarkable, with a heart rate of 75 bpm, blood pressure of 120/80 mmHg and oxygen saturation of 97%. Cardiopulmonary auscultation was normal, with the exception of some crepitations in the lung bases. The neurological examination was largely normal, but the diver was unstable in the Romberg maneuver and unable to stand. The ECG indicated the presence of incomplete right bundle branch block. The biological assessment revealed no significant abnormalities, aside from hyperleukocytosis at 16,800 GB/L, an LDH level of 296 UI/L (normal value < 225), and a myoglobin level of 765 microg/L (<58). The patient’s glycemia was 5.7 mmol/L, the ultrasensitive troponin T was negative (<13), the NT pro BNP was 52 ng/L (28–27), the D-dimer was 0.42 microg/mL (0–0.5), and the CRP was 0.8 mg/L.

The patient was initially assessed with a CT scan of the chest to look for signs of pulmonary hypertension, given the initial loss of consciousness associated with rapid ascent to the surface. The diagnosis of gas embolism due to pulmonary barotrauma is not supported by the available evidence. The patient’s neurological condition then deteriorated into a state of agitation, which made it impossible to perform the examination safely and reliably.

MRI of the brain was performed after sedation. The initial MRI on day 1 was performed according to a “stroke” protocol with axial slices in T2 gradient echo, FLAIR, diffusion-weighted imaging (DWI) and phase contrast on the polygon of Willis. MRI showed cortical and subcortical, supratentorial and subtentorial FLAIR hyperintensities with cortical oedema in the right frontal, right medial parietal, bilateral polar temporal, bilateral and symmetrical cerebellar and internal capsule regions. These anomalies were mainly located in the posterior cerebral area.

These images reflected peri-injury vasogenic oedema suggestive of capillary hyperpermeability. There was no evidence of ischaemic lesions (absence of diffusion hypersignal or apparent diffusion coefficient decrease in the affected areas) or haemorrhagic lesions on T2 gradient echo.

Phase contrast sequence over the polygon of Willis showed no evidence of vascular thrombus, cerebral arterial malformation or vascular occlusion without sign of cerebral vasoconstriction.

The ventricular system appeared normal and the venous sinuses were permeable on spontaneous contrast.

At 7:58 p.m., a therapeutic recompression table with oxygen at 2.8 atm abs for 2.5 h was performed for a total of two and a half hours, combined with adjunctive treatment with methylprednisolone 1 mg/kg body weight and acetylsalicilic acid 500 mg, while the patient remained on mechanical ventilation.

After therapeutic recompression, the patient was transferred to the intensive care unit overnight and extubated approximately 2 h later.

The next morning the patient was calm and alert with a Glasgow score of 15; interview revealed temporo-spatial disorientation, difficulty following given instructions; vocabulary range was limited with verbal repetition, word loss and difficulty with ideation. Clinical examination revealed an unstable Romberg with leftward deviation, increased sustentation polygon on gait, generalised weakness and difficulty walking unaided. There was no spontaneous nystagmus or vestibular syndrome. The motor and sensory examination was unremarkable, as was the rest of the clinical examination.

Given these persistent symptoms, which did not completely resolve after the first therapeutic recompression, it was decided to continue treatment with hyperbaric oxygen therapy using two Heliox recompression tables at 2.8 atm abs, then 100% O_2_ at 9 m (Heliox B18 table), in accordance with our protocol for the treatment of severe neurological DCS (Simonnet et al., 2023). The patient then received additional daily sessions of 80 min HBOT at 2.5 atm abs (100% O_2_) for 9 days.

Clinical examination after each session showed progressive regression of cognitive and then vestibular dysfunction, leading to normalisation of the patient’s clinical condition during the final days of treatment.

**FIGURE 2:**
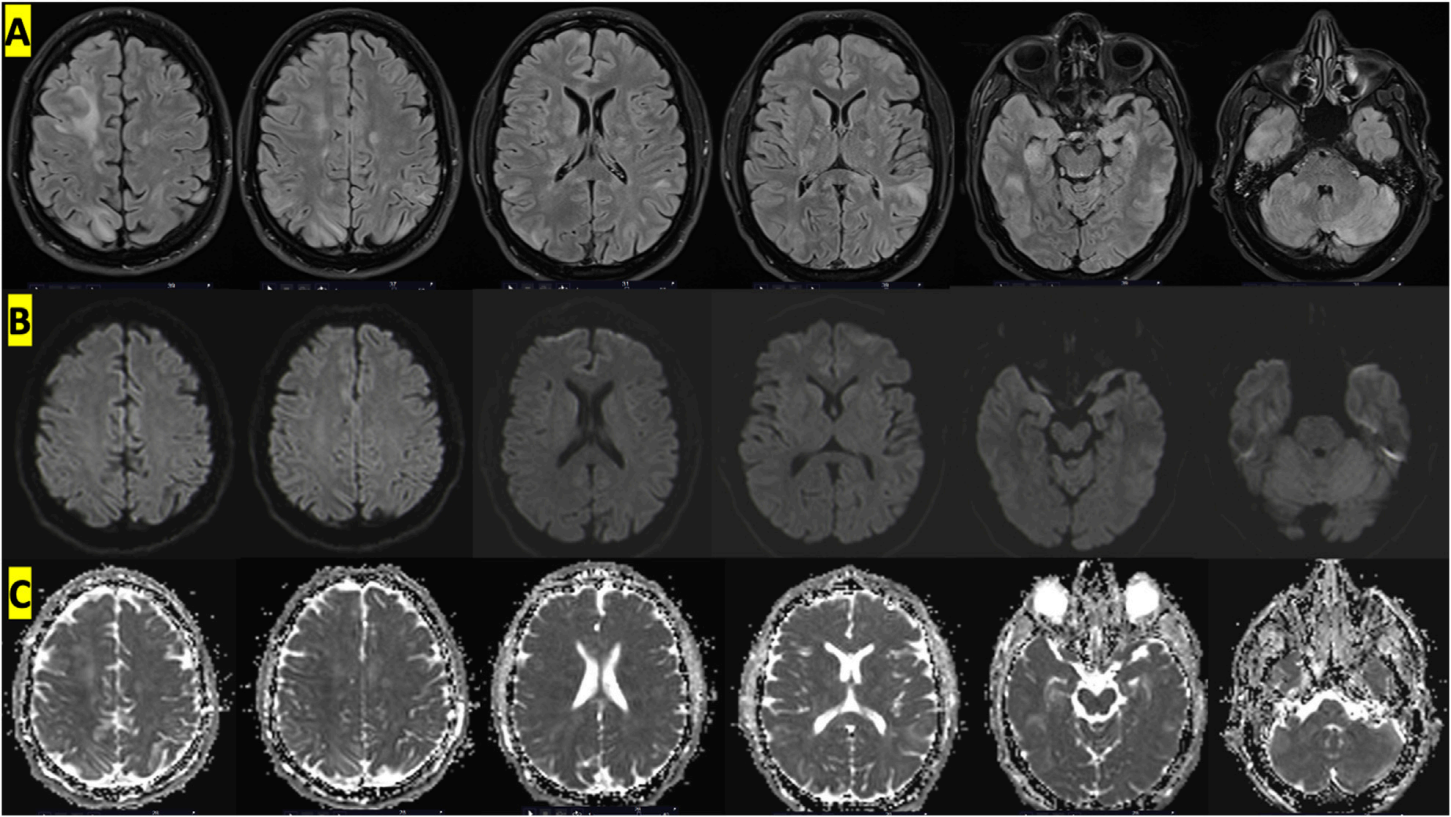
Cerebral MRI with an appearance suggestive of vascular edema showing diffuse cortico-subcortical, supratentorial and subtentorial involvement on FLAIR sequences. **(A)** T2 FLAIR sequences showing hyperintense signals at different levels, **(B)** absence of hyperintensities in DWI sequences and **(C)** increased value on ADC map.

An additional MRI was then performed on day 2 with a perfusion sequence and a brain injection sequence to try to characterise the lesions. On the latter, the lesions appeared more prominent and extensive in the right hippocampus and corpus callosum, with discrete cortical contrast enhancement of the right frontal abnormalities, without perfusion abnormalities or neoangiogenesis, associated with a blood-brain barrier rupture (Figure 2). There was still no evidence of ischaemic lesions or haemorrhagic remodelling.

After several sessions of hyperbaric oxygen therapy, a 1-week follow-up MRI was performed. This showed a clear reduction in the size of the various lesions previously visualised, with disappearance of cerebellar, hippocampal, corpus callosum and parietal lesions. Only signal abnormalities remain, with a right frontal cortico-subcortical Flair hypersignal, but these were also much less extensive.

Additional tests were performed:

- Lumbar puncture performed on day 2: hyperproteinorachia 0.76 g/L, normoglycorachia, IgG index = 0.39, negative iso-focalisation, 39 erythrocytes and 3 leukocytes.
- EEG performed on day 2: normal background activity, diffuse, with bursts of slow waves projecting mainly forwards. No visible critical or paroxysmal activity;
- Biological results within the first 7 days:
  - Spontaneously regressive neutrophilic hyperleukocytosis, with no associated biological inflammatory signs. Blood ionogram, liver function tests and renal function normal;
  - Absence of coagulopathy and disseminated intravascular coagulation;
  - Negative immune dysfunction panel and infectious disease panel;
- Transcranial Doppler search for right-left shunt performed 7 days later was negative.
- Positron emission tomography performed 10 days later showed residual right dorsolateral frontal fixation, consistent with control MRI;
- Vestibulo-nystagmogram performed 10 days later showed a left peripheral vestibular deficit estimated at 45% on caloric tests.

## Discussion

In this context of prolonged deep BH diving a with surface disturbances of consciousness, an initial diagnosis of hypoxic syncope complicated by a convulsive state could have been evoked. However, the clinical course and imaging revealed an acute encephalopathy in favour of Taravana syndrome. This accident was associated with other sequelae: the thoracic CT scan showed ground-glass opacities consistent with minimal immersion pulmonary oedema and vestibular damage, probably of barotraumatic origin, with a left peripheral vestibular deficit found by vestibulo-nystagmogram.

All these symptoms were compatible with “extreme” freediving, with very fast ascent and descent rates on an underwater scooter at 40 m depth for 3-minute work periods. Clinical cases of Taravana syndrome associated with the use of underwater scooters have been described in the past (Grandjean and Fanton, 1996; Blogg et al., 2023).

The rate of descent and ascent were particularly high in our case, which was a risk factor for both barotrauma and inert gas loading. A review of the scooters currently available for purchase online revealed that the majority have a maximum speed of between 50 and 170 m per minute. Using these maximum speeds can be dangerous for freedivers during the ascent phase, especially since some models are designed for dives to depths of up to 50 m. We believe it is essential that national and international diving organisations and manufacturers specify speed limits during ascent phase that must not be exceeded in order to comply with decompression procedures.

This case study prompted further investigation into the underlying mechanisms of Taravana syndrome. The imaging study suggested the formation of cerebral bubbles *in situ* and vasculopathy, a mechanism previously observed in other cases (Matsuo et al., 2014; Guerreiro et al., 2018; Sánchez-Villalobos et al., 2022). We subsequently reviewed the primary pathophysiological hypotheses that explain the onset of Taravana syndrome.

In Taravana syndrome, deep dives (over 15 m, rarely reaching 50 m or more), immersion times between 1 min 20 s and 2 min, and relatively short intervals between each apnea, of the order of a few minutes (3–6 min on average) were initially considered as risk factors.

These early descriptions of neurological symptoms suggested mainly a cerebral involvement, with paralytic symptoms (monoplegia, hemiplegia, etc.) as well as “psychic” symptoms such as frontal syndrome, memory and ideation disorders, attention and speech that may become logorrheic and incomprehensible (Bagnis, 1968; Cross, 1965).

More recently reviewed studies repeat the same risk factors, associating deep BH diving with immersion times of the order of a minute or more, sometimes with very rapid ascent rates and rather short surface intervals (Lemaître et al., 2009; Tetzlaff et al., 2016; Blogg et al., 2023).

Other cases have been described in single, very deep dives by record-breaking freedivers. Fitz- Clarke et al. have identified two cases of Taravana that were recorded out of 24 divers who made a series of 192 BH dives to a depth of 100 m or more (Fitz-Clarke, 2009).

### Origin of Taravana: main theories

One of the theories often reported is that Taravana syndrome is a specific form of DCS with the formation of venous gas bubbles passing via a right-to-left shunt through a foramen ovale (PFO) (Gempp and Blatteau, 2006) or an intrapulmonary arteriovenous anastomosis (Kohshi et al., 2021). Hypoxia during BH diving could play a role by promoting the recruitment of an intrapulmonary arteriovenous anastomosis, allowing venous nitrogen bubbles to pass into the arterial circulation (Schipke and Tetzlaff, 2016).

However, venous circulating bubbles after BH diving were rarely detected and usually in small amounts (Spencer and Okino, 1972; Boussuges et al., 1997; Lemaître et al., 2014; Cialoni et al., 2016; Barak et al., 2020).

Others have suggested that bubbles may form directly in the cerebral arteries (Goldman and Solano-Altamirano, 2015; Bosco et al., 2020). For Muth, very fast ascent rates would lead to the formation of intra-arterial bubbles at the origin of a cerebral gas embolism (Foster et al., 2016). Mathematical modelling of the birth and evolution of these bubbles in the arterial system would be possible, both for single but very deep BH diving as and for shallower but repeated BH diving as, allowing these bubbles to embolize the cerebral capillaries (Goldman and Solano-Altamirano, 2015). High cerebral blood flow rates, which affect uptake and elimination rates, may also play a role in the transient nature of symptoms sometimes reported in Taravana syndrome.

It is currently accepted that decompression bubbles originate from very small persistent gaseous phases called gas micronuclei (Blatteau et al., 2006). These gas micronuclei could actually be nanobubbles that developed mainly on hydrophobic sites that can be identified on endothelial surfaces and especially in the brain (Arieli and Marmur, 2017; Arieli, 2019a; Arieli, 2019b). These nanobubbles could therefore feed decompression bubbles in distal arteries or capillaries in the brain, in the form of autochthonous bubbles. Foster et al. suggested that vasogenic oedema seen on Taravana MRI was more consistent with blood-brain barrier damage than gas bubble embolism favoured by intrapulmonary shunting (Foster et al., 2016).

For Kohshi et al. small amounts of intravascular bubbles may be the first step of neurological DCS in BH divers. They may form thrombi or microparticles and can be responsible for cerebral vascular autoregulation dysfunction with a blood-brain barrier breakdown (Barak et al., 2020; Kohshi et al., 2021).

Other factors may be involved in the occurrence of Taravana. The main hypotheses cited in a recent review are as follows (Foster et al., 2016):

- Disruption of cerebral blood flow due to hypoxia and hypercapnia;
- Bubble passage by mechanical effect: as blood movement during ascent is not as fast as pulmonary expansion, this would lead to bubbles entering the arterial circulation;
- Pulmonary barotrauma as a potential trigger for venous gas embolism, which would then feed the cerebral circulation via an arterio-venous shunt;
- Biological factors associated with the presence of intravascular bubbles, including endothelial changes, blood-brain barrier disruption, complement activation, inflammatory events, and platelet activation.

In addition, recurrent Taravana syndrome has been described in a scuba diver whose evaluation revealed hyperhomocysteinemia, which is implicated in the mechanism of stroke onset (Accurso et al., 2018).

The multiplicity of pathophysiological hypotheses and the complexity of the mechanisms induced by immersion, BH diving and mechanical stress suggest a multifactorial origin for the occurrence of the Taravana syndrome in BH divers.

### Proposed main mechanism

In our description, the diffuse multifocal brain damage seen on MRI was consistent with microbullar encephalopathy secondary to local bubble formation (“autochthonous bubbles”) and not related to bubbles of aeroembolic origin. The absence of pulmonary barotrauma and left-to-right shunting was consistent with this hypothesis of bubble formation in brain tissue saturated by hyperbaric exposure at significant depths. The appearance of the initial symptoms several minutes after exiting the water was suggestive of DCS. Conversely, hypoxic unconsciousness typically occurs in open water or immediately upon surfacing.

Brain tissue, consisting mainly of adipose tissue, is a tissue that rapidly saturates with nitrogen when exposed to high partial pressures. The rapid rate of ascent experienced by the diver does not allow this so-called fast tissue to remain below the threshold of critical supersaturation, thus creating the bed for the birth of pathogenic bubbles, locally responsible for complex and self-sustaining immune phenomena, the bubble being recognised as a foreign object.

Initial MRI showed cortical and subcortical, supratentorial and subtentorial FLAIR hyperintensities with cortical oedema in the right frontal, right medial parietal, bilateral polar temporal, bilateral and symmetrical cerebellar and internal capsule regions. However, the results of the diffusion-weighted imaging sequences did not indicate the presence of hyperintensities, with normal values on the apparent diffusion coefficient (ADC) map. This suggests that there are no ischemic lesions present (Kidwell et al., 2000). This was a crucial point in the analysis of the proposed mechanisms. The discrepancy between the diffusion and FLAIR sequences indicated that the mechanism was not ischemic or embolic. Therefore, the hypothesis of a paradoxical embolism caused by arterial passage of nitrogen bubbles via a right-to-left shunt was not considered. Cerebral lesions resulting from arterial gas embolism typically present with a radiological appearance analogous to that of a stroke (restricted diffusion appearing as hyperintensity on DWI with low values on the ADC map) (Kamtchum Tatuene et al., 2014). In light of the presented case, the most probable cause appears to be the formation of bubbles directly within the cerebral structures, particularly in the capillaries. This would explain the edema and capillary hyperpermeability observed by the radiologists. It is worth noting that our MRI analysis was consistent with other reported cases of Taravana, including cerebral MRI (Matsuo et al., 2014; Guerreiro et al., 2018; Sánchez-Villalobos et al., 2022). Furthermore, it is worth noting that bilateral and posterior lesions of this nature are relatively uncommon. Our analysis suggests that the diffuse brain damage observed in this case may have been caused by the rapid ascent rates experienced after the deep dives discussed above.

Another important point to emphasize is that the bilateral, posterior and sometimes symmetrical lesions observed on MRI were highly suggestive of a posterior reversible encephalopathy syndrome. This syndrome is particularly common in hypertensive crises leading to encephalopathy. Other conditions such as pre-eclampsia, chemotherapy, sepsis, chronic renal failure and autoimmune diseases can cause this syndrome (Fugate and Rabinstein, 2015; Saad et al., 2019; Tetsuka and Ogawa, 2019). Seizures, confusion and loss of balance are classic signs of PRES. The typical presentation shows diffuse cortical, subcortical and deep lesions, preferably in the parietal and occipital lobes, but which may also involve the frontal and temporal lobes and, less commonly, the cerebellar hemispheres. Bilateral and symmetric lesions are very characteristic, although asymmetric involvement occurs in one-third of cases (Fugate and Rabinstein, 2015; Saad et al., 2019; Tetsuka and Ogawa, 2019). In our case, the lesion pattern, distribution and evolution were highly suggestive of PRES (Kastrup et al., 2015).

The pathophysiological basis of PRES remains controversial. Hypertension-induced autoregulatory failure with subsequent hyperperfusion has long been the dominant hypothesis. However, other theories currently suggest vasoconstriction-induced hypoperfusion with subsequent local ischaemia, blood-brain barrier breakdown and vasogenic oedema as the underlying mechanism (Anderson et al., 2020).

It is interesting to discuss the two mechanisms that could explain the occurrence of PRES in BH diving. Some authors have reported the possibility of particularly high blood pressure during freediving in non-hypertensive subjects (Ferrigno et al., 1997). The occurrence of hypertensive surges during freediving could have contributed to the formation of cerebral vasogenic oedema in certain subjects. However, in the reported case, the diver had no history of hypertension and his blood pressure was normal on admission to hospital.

Another hypothesis, already mentioned, seems more likely to explain the occurrence of Taravana. This is the formation of autochthonous bubbles directly in the cerebral structures, particularly in the capillaries, during decompression. The growth of bubbles within the cerebral capillaries could lead to local immuno-inflammatory reactions with damage to the blood-brain barrier, responsible for extracellular oedema affecting mainly the white matter via fluid leakage from the capillaries. The number and location of autochthonous bullae and the extent of immuno-inflammatory responses may explain the inter-individual variability and clinical forms of Taravana.

After thorough examination, we have determined that this case of Taravana aligned with the characteristics of microbullar encephalopathy. We believe that the formation of bubbles *in situ* in the capillaries that open the blood-brain barrier is the triggering event. This results in a loss of cerebral autoregulation with vasodilatation and hyperperfusion, which is responsible for vasogenic edema. It should be noted that perforating arteries and vertebrobasilar arteries have less developed protective perivascular sympathetic innervation than arteries of anterior localization. This explains the preferential posterior localization of anomalies. One hypothesis to account for blood-brain barrier rupture in PRES is the alteration of endothelial tight junctions. It is highly probable that microbubbles played an important causal role here.

## Conclusion

On the basis of the MRI data, we hypothesised that the Taravana syndrome described in the present case, with very rapid ascent rates associated with the underwater scooter, corresponded to a diffuse microbullar encephalopathy, i.e., PRES with vasogenic oedema not associated with an ischaemic and embolic mechanism.

We believe that the initial mechanism was the *in situ* formation of autochthonous bubbles in the cerebral capillaries. Cerebral tissue is a “fast” tissue, rapidly saturating with nitrogen, and the rapid rate of rise does not allow it to remain below the critical supersaturation threshold, which favours the growth of bubbles *in situ*, locally responsible for the alteration of the capillary barrier and immuno-inflammatory phenomena.

This hypothesis needs to be confirmed in other cases by systematically performing MRI with diffusion sequences.

It is recommended that users of underwater scooters be provided with preventive messages that rigorously limit ascent speeds in freediving.

## Data Availability

The original contributions presented in the study are included in the article/Supplementary Material, further inquiries can be directed to the corresponding author.

## Supplementary material

SUPPLEMENTARY MATERIAL Film showing the freedive performed with an underwater scooter on the injured diver just before the onset of symptoms on return to the surface.
